# Effects of combination mixture of *Cannabis sativa* with psychoactive drugs on adenosine deaminase, ectonucleosides enzymes, redox status, cognition and hemodynamic function in male rats

**DOI:** 10.1002/alz.085050

**Published:** 2025-01-09

**Authors:** Olufunke Florence Ajeigbe, Ayokunle Olubode Ademosun, Ganiyu Oboh, Bodun Oluwaseun Lawrence

**Affiliations:** ^1^ Elizade University, Ondo State, Akure Nigeria; ^2^ Federal University of Technology Akure, Ondo State, Akure Nigeria

## Abstract

**Background:**

The effect of high consumption of psychoactive substances of codeine (CDE), tramadol (TMD), and *Cannabis sativa* (CNB) as concoction has been associated with altered brain cognitive and neurochemical functions. However, the understanding of the complex mechanism behind the intake of *Cannabis sativa* co‐administration with tramadol and codeine on both cardiac and brain function, neurotransmitters, purinergic, and antioxidant enzymes activities in the brain and heart of rats remains unreported.

**Method:**

The measure of cognition using morris water maze (MWM) and Y‐maze tests, hemodynamic parameters namely systolic blood pressure (SBP) and heart rate (HR), acetylcholinesterase (AChE), butyl‐cholinesterase (BCHE), adenosine deaminase (ADA), arginase, catalase (CAT), superoxide dismutase (SOD) enzymes’ activities, reduced glutathione (GSH) and malondialdehyde (MDA), nitric oxide (NO) levels, in the brain and heart of CNB, TMD, and CDE exposed rats was done. Forty‐eight male Wistar rats were divided into 8 groups, n = 10. Group 1 was the control group, groups 2, 3, and 4 received oral doses of CDE (2 mg/kg bw), TMD (10 mg/kg bw), and CNB (200 mg/kg bw), while groups 5, 6, 7, and 8 received oral doses of CDE+TMD, CNB+ TMD, CNB+CDE, and CNB+TMD+CDE for a 28‐day period.

**Result:**

Coadministration of CNB, TMD, and CDE was seen to led to the significant decrease in cognitive function, acetyl‐cholinesterase (AChE), butyl‐cholinesterase (BChE), monoamine oxidase (MAO) enzyme activities, and antioxidant enzyme activities in rats' brains when compared against control rats (P< 0.05). Interestingly, CNB, TMD, and CDE co‐administration led to significant decrease in SBP and HR whereas, the activities of ectonucleosides (NTPdase), adenosine deaminase (ADA), and malondialdehyde levels produced in the brain and heart were significantly elevated (P< 0.05) in rats.

**Conclusion:**

Therefore, coadministration of CNB, TMD, and CDE led to neurotransmitter poisoning and hypotensive potential in rats which are a notable mechanism of actions in the understanding of the drug development and synergistic neurotoxicity effects of CNB, TMD, and CDE on rats' hemodynamic and cognitive functions, as well as cholinergic, purinergic, and antioxidant systems.

